# Novel Numerical Characterization of Protein Sequences Based on Individual Amino Acid and Its Application

**DOI:** 10.1155/2015/909567

**Published:** 2015-02-02

**Authors:** Yan-ping Zhang, Ya-jun Sheng, Wei Zheng, Ping-an He, Ji-shuo Ruan

**Affiliations:** ^1^Department of Mathematics, School of Science, Hebei University of Engineering, Handan 056038, China; ^2^Graduate School at ShenZhen, Tsinghua University, Guangdong 518055, China; ^3^College of Mathematical Sciences and LPMC, Nankai University, Tianjin 300071, China; ^4^Department of Mathematics, College of Science, Zhejiang Sci-Tech University, Hangzhou 310018, China

## Abstract

The hydrophobicity and hydrophilicity of amino acids play a very important role in protein folding and its interaction with the environment and other molecules, as well as its catalytic mechanism. Based on the two physicochemical indexes, a 2D graphical representation of protein sequences is introduced; meanwhile, a new numerical characteristic has been proposed to compute the distance of different sequences for analysis of sequence similarity/dissimilarity on the basis of this graphical representation. Furthermore, we apply the new distance in the similarities/dissimilarities of ND5 proteins of nine species and predict the four major classes based on the dataset containing 639 domains. The results show that the method is simple and effective.

## 1. Introduction

It is becoming increasingly important to accurately predict structure and function of proteins because there is an increasing amount of protein sequences collected. Now, many methods have been proposed to gain the additional information or knowledge about the sequence. Graphical representations have become an effective aid in understanding numerical characterizations of biological sequences. One method of creating a graphical representation of a biologic sequence is to create a mapping from the sequence of amino acids or bases, in increasing sequence order, to a numeric characterization of a property of the amino acid or base. According to the numerical characterizations, we can further analysis and research of biological sequences.

The graphical technique was firstly proposed by Hamori [1] for representation of DNA sequences. And then many graphical representations of DNA sequences were provided, for example, 2D, 3D, and other graphical representations of DNA sequences [2–10].

Graphical representation of protein sequences has emerged recently [11–21]. On the basis of the genetic code, Randić et al. [11–14] gave some graphical representations of protein sequences. Recently, many graphical representations of protein sequences are generated according to the physicochemical properties of 20 AAs [15–21].

In order to have a more intuitive understanding about the biological characteristics implied in the sequence and analyze the similarity/dissimilarity of the protein sequences, Randić and others [22–26] proposed many numerical characterizations, such as *M*, *D*, *M*/*M*, *L*/*L*(*D*/*D*), *L* ^*k*^/*L* ^*k*^ matrix. For example, *M*/*M* matrix is the quotient of the Euclidean distance and the Graph distance between points in the curve; *L*/*L*(*D*/*D*) represents quotient of the Euclidean distance and the sum of distances between a pair of points in the curve. Furthermore, these different characteristic invariants were applied to compare the similarities of biological sequences. However, the numerical characterization methods require a great amount of calculation and lose some information of sequences. So many simple and direct methods were proposed in order to solve complex problems in the sequence alignment. For instance, Randić et al. [27, 28] and He et al. [19] directly apply the generating graphical representation of protein sequences to compare the similarities/dissimilarities of the protein sequences of different species.

In this paper, a 2D graphical representation of protein sequences is introduced based on the hydrophobicity and hydropathy index. According to the graphical representation, a new numerical characteristic has been proposed to compute the distance of different sequences for analysis of sequence similarity/dissimilarity. Then, we use the new numerical characteristic of graphical representation to analyze the similarities/dissimilarities of ND5 proteins of nine species. For illustrating the utility of our method, the correlation analysis has been provided to compare between our results and the results based on the other graphical representations with the ClustalW's results. Furthermore, we utilize our method to predict protein structural class, the prediction accuracy of All-*β*, *α* + *β* class and the overall accuracy have obviously improvement. The result indicates that EH and Hp indexes have important function when the primary sequence folds into secondary structure; it also indicates that our method is simple and effective.

## 2. The Graphical Representation of Protein Sequences

The hydrophobicity and hydrophilicity of AAs in a protein play an important role in its folding and its interaction with the environment and other molecules, as well as its catalytic mechanism [29]. Based on the hydrophobicity (EH) [30] and hydropathy (Hp) [31] index which were considered by Kurgan and Chen [32], we introduce a graphical representation of proteins to analyze the evolutionary relationships of the protein sequences and predict the structural class from the primary sequences. At first, we consider mapping of each AA, as follows:

(1) EHt1=EHt0−∑i=120EHt020,Hpt1=Hpt0−∑i=120Hpt020,

where the EH_*t*_^0^ and Hp_*t*_^0^ (*t* = 1,2,…, 20) are the original EH and Hp values of 20 AAs which are listed in columns 3 and 4 of Table 1, respectively. Based on (1), the 2D-Cartesian coordinates of 20 AAs are listed in columns 5 and 6 of Table 1, respectively. Because the slope decides the direction of a curve, we use an equation to construct a 2D graphical representation for each protein sequence, as follows.

For a protein sequence *S* = *s*_1_ *s*_2_ ⋯ *s*_*n*_, inspect it by stepping one AA at a time. For step *i*  (*i* = 1,2,…, *n*), a 2D space point *P*_*i*_(*x*_*i*_, *y*_*i*_) can be constructed as follows:

(2) xi=i,yi=Hpt1EHt1.

Let *P*_0_(*x*_0_, *y*_0_) = (0,0). When *i* runs from 1 to *n*, we obtain a series of points *P*_1_, *P*_2_,…, *P*_*n*_, connecting the adjacent points in turn; a 2D zigzag curve that contains *n* + 1 points can be obtained.

**Figure 1:**
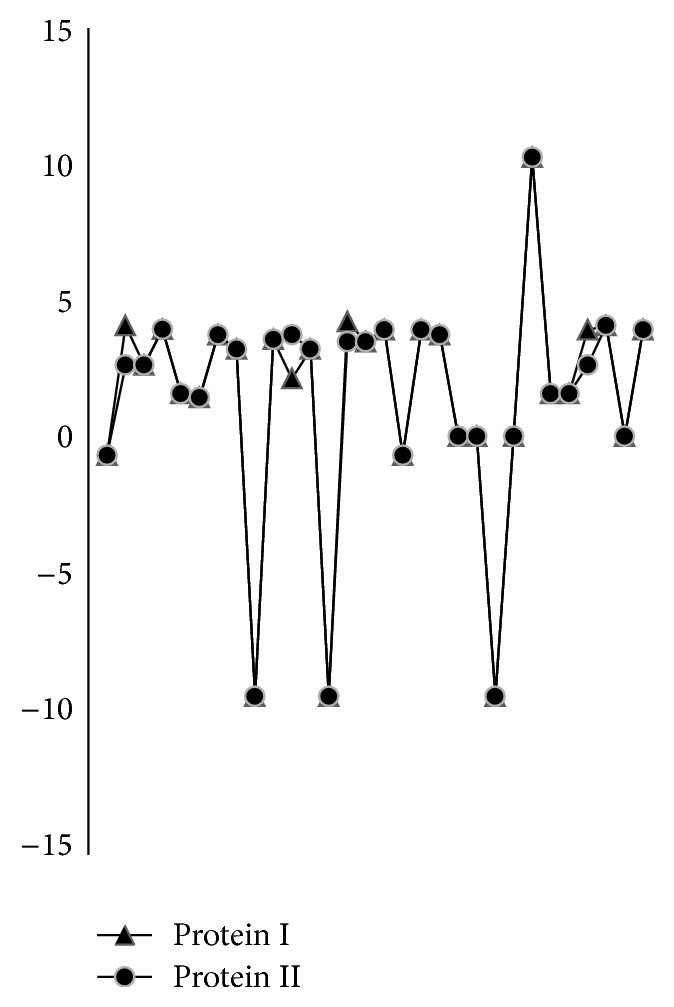
The two curves of protein sequences I and II in the coordinate value.

As an example, the 2D graphical representations of the two short protein segments of Saccharomyces cerevisiae [27] are plotted in Figure 1 to illuminate our approach.

In the curve, *x*-, *y*-coordinate values represent the positions of AAs in the sequence and the direction of the curve, respectively. And we find that the protein sequences I and II are generally similar except four AAs no matching.

## 3. The New Distance Metrics of Two Sequences

In order to have a more intuitive understanding about implied biological characteristics in the sequence and analyze the similarity/dissimilarity of different protein sequences, many authors proposed different characteristic invariants in different matrices, such as the *D*, *E*, *L*/*L*, *M*/*M*, *L* ^*k*^/*L* ^*k*^ matrices [22–26]. However, the numerical characterization methods require a great amount of calculation and may lose some information of sequences. Therefore, some researchers used the cumulative distance of every point to present the distance of the sequences [20, 27, 28]. These numerical characterizations can avoid losing some information of the protein sequences.

We define the distance metrics between sequences *S*_1_ and *S*_2_ by (3) to compute the similarity of sequences:

(3) D(S1−S2)=∑i=1l1yS1i−yS2il1if   l1=l2∑i=1l2yS1i−yS2i+∑i=l2+1l1yS1il1if   l1>l2,

where *l*_1_, *l*_2_ denote the lengths of two sequences *S*_1_ and *S*_2_; *y*_*S*_1__, *y*_*S*_2__ are their *y*-coordinate values, respectively. This distance eliminates reflection of no equal length sequences, so the numerical characterization is more effective.

## 4. The Similarity/Dissimilarity Analysis of Nine ND5 Proteins

We use the novel quantitative description of the graphical representation of protein sequences to analyze the similarities/dissimilarities of ND5 proteins of nine species (Human (AP_000649, 603aa), gorilla (NP_008222, 603aa), pygmy chimpanzee (pygmy) (NP_008209, 603aa), common chimpanzee (common) (NP_008196, 603aa), fin whale (NP_006899, 606aa), blue whale (NP_007066, 606aa), rat (AP_004902, 610aa), mouse (NP_904338, 607aa), and opossum (NP_007105, 602aa)).

The distances among ND5 proteins of nine species are computed based on (3), and their similarities/dissimilarities are listed in Table 2. The smaller distance represents the two species are more similar. Observing Table 2, we find the fin whale-blue whale is the most similar. The human, gorilla, pygmy, and common are also similar, and the rat and mouse are similar. Furthermore, we find the opossum is the dissimilar to the other eight species. And we obtain the human is more similar to pygmy and common than human and gorilla. These results about the similarity are consistent with the known fact of evolution and reduce the computational complexity.

**Figure 2:**
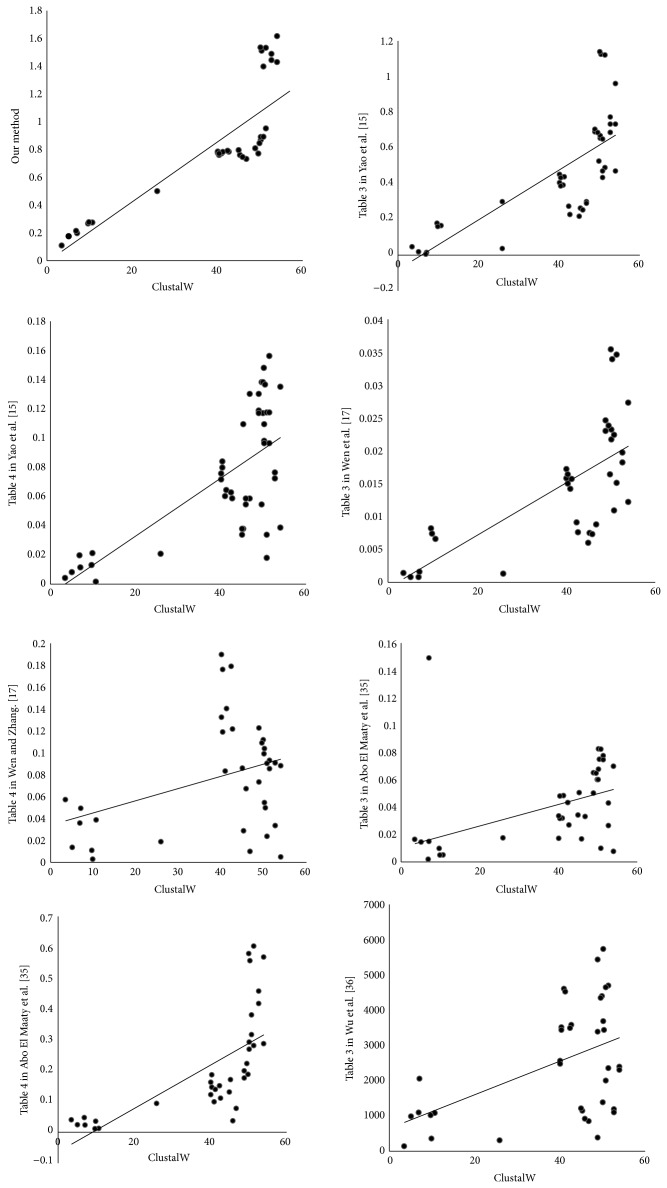
The correlation analysis between ClustalW and other methods.

To illustrate the effectiveness of our method, the ClustalW is used to compute the similarity of sequences and construct the phylogenetic tree [34]. ClustalW is a multiple sequence alignment program for biological sequences, which attempts to calculate the best match for the selected sequences and lines them up so that the identities, similarities, and differences can be observed. Then, the distance matrix for ND5 proteins of nine species is calculated by ClustalW and listed in Table 3. In order to illustrate the effectiveness of our method, we give the scatter plot of correlation analysis from element by element of Tables 2 and 3. If the points are all round the trend line, this shows that the correlation is better between our method and ClustalW. Furthermore, the scatter plots of correlation analysis are obtained about the results of Yao et al. method [15], Wen and Zhang method [17], Abo El Maaty et al. method [35], and Wu et al. method [36] with the distance matrix of Table 3. Observing Figure 2, our method is better than other graphical representation approaches of proteins.

## 5. The Prediction of Structural Class Using *k*-NN Algorithm

Protein function, regulation, and interactions can be learned from their structure [37, 38], which promotes development of novel methods for the prediction of the protein structure. And knowledge of protein structure plays an important role in molecular biology, cell biology, pharmacology, and medical science.

Protein secondary structural is generally classified into four structural classes: all-*α*, all-*β*, *α*/*β*, and *α* + *β*. The all-*α* and all-*β* classes represent structures that contain mainly *α*-helices and *β*-strands, respectively. The *α*/*β* and *α* + *β* classes include both *α*-helices and *β*-strands where the *α*/*β* class consists of mainly parallel *β*-strands and *α* + *β* class includes antiparallel strands. We obtain that the dataset includes 640 domains that share sequence identity below 25% [33] in http://biomine.ece.ualberta.ca/Structural_Class/SCEC.html. In this paper, we use the dataset that only includes 639 protein domains deleting a wrong domain.

In this work, the *k*-Nearest Neighbor (*k*-NN) classifiers algorithm is used to predict the structural class. The *k*-NN algorithm is the simplest among those used in machine learning and can determine the attribute of a query point by taking the weighted average of the *k*-NN to the point, and as such is a highly effective inductive inference method [39]. Given a sequence *S*, we calculate the distance metrics of sequence *S* with other sequences and select the *k*-nearest sequences. The distance metrics *D*(*S*_1_ − *S*_2_) between two sequences *S*_1_ and *S*_2_ are calculated using (3). In the *k* sequences, we use the *N*1, *N*2, *N*3, *N*4 to indicate the numbers of sequences which belong to all-*α*, all-*β*, *α*/*β*, and *α* + *β* class, respectively. If the *N*1 or (*N*2 or *N*3 or *N*4) is the maximum, sequence *S* is, respectively, predicted for all-*α*, all-*β*, *α*/*β*, and *α* + *β* class. According to the calculation process, we list the performance results of our method using the jackknife test when *k* = 29 in Tables 4 and 5 (i.e., to say *N*1 + *N*2 + *N*3 + *N*4 = 29).

The following evaluation of the predicted results used several quality measures in this work, including the prediction accuracy (ACC), sensitivity, specificity, and Matthews correlation coefficient (MCC). In the section, the ACC was used to evaluate the results of our method and other published approaches:

(4) Accuracy=TP+TNTP+TN+FP+FN,Sensitivity=TPTP+FN,Specificity=TNTN+FP,MCC=TP×TN−FP×FN(TN+FN)×(TN+FP)×(TP+FN)×(TP+FP),

where TP and TN are the numbers of correctly classified sequences of positive and negative samples, respectively. FP and FN are the numbers of incorrectly classified sequences of negative and positive samples, respectively. The simple and intuitive of ROC curve is given that can accurately reflect a specificity and sensitivity analysis method and is the comprehensive representation of the test accuracy. Meanwhile, the area under the ROC curve (AUC) is given to evaluate the predicted probabilities.

**Figure 3:**
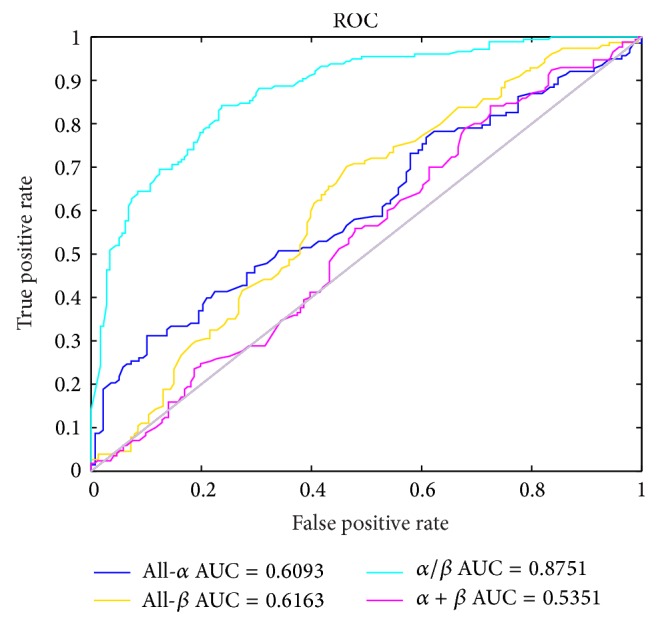
The ROC curve about the four classes (all-*α*, all-*β*, *α*/*β*, and *α* + *β*) and AUC values, respectively.

Observing Table 4, the results indicate that the overall prediction accuracy with our method achieves 60.82% in the 639 domains, which is the highest among the compared methods, including IB1, C4.5, Naive Bayes, logistic regression [33], and Liao's method [20]. In Chen's article [33], the authors declared that *α* + *β* class was the most difficult to predict than the other three structural classes. However, the prediction accuracy of *α* + *β* has evidently improved using our method. And the all-*β* class and overall accuracy are also higher than other methods. The result demonstrate that EH and Hp index possess very important function when the primary sequence folds into secondary structure especially in the *α* + *β* class. Furthermore, using our method, the other performance values and the ROC curves by utilizing individual four classes and corresponding AUC values are given in Table 5 and Figure 3, respectively. Observing Table 5, the predictions for the *α*/*β* class have higher quality with 65.25% for sensitivity, 91.58% for specificity, and 50.36% for MCC. In Figure 3, the AUC values for each of the four classes are above 0.5 (for random predictions). Although the overall prediction accuracy with our method is lower than the method of SVM [33], our approach is simpler and less time consuming.

## 6. Conclusions

The hydrophobicity and hydrophilicity of AAs play an important role in folding for secondary structure. Based on the two physicochemical indexes, a 2D graphical representation of protein sequences is proposed in the paper. This graphical representation of protein sequences has the better visibility and can reflect more information of protein sequences. In order to obtain the intuitive understanding of sequences implying biological characteristics and make the similarity comparison conveniently, a new distance is suggested based on the graphical representation of protein sequences. We firstly apply the new distance to analyze the similarities/dissimilarities of ND5 proteins of nine species, and correlation analysis is given to compare our results and other graphical representations with ClustalW's result. Furthermore, using the new distance of graphical representation, the four major classes are predicted based on the dataset containing 639 domains that share sequence identity below 25%. The prediction result shows that the method can improve the prediction accuracy for All-*β*, *α* + *β* class and the overall accuracy. In particular, using our method can evidently improve the prediction accuracy of the *α* + *β* class. The result demonstrates that EH and Hp index have important function when the primary sequence folds into secondary structure. The calculation methodology is more simple, convenient, and fast. In addition, the method can be extended to other physicochemical properties of amino acids and will be useful to study and solve some bioinformatics problems.

## Figures and Tables

**Table 1:** The EH_*t*_^0^ and Hp_*t*_^0^ values of 20 AAs and their coordinates in the 2D-Cartesian derived from (1).

Amino acid	Code	EH^0^	Hp^0^	EH^1^	Hp^1^
Alanine	A	0.62	1.8	0.62	2.29
Cysteine	C	0.29	2.5	0.29	2.99
Aspartate	D	−0.9	−3.5	−0.9	−3.01
Glutamate	E	−0.74	−3.5	−0.74	−3.01
Phenylalanine	F	1.19	2.8	1.19	3.29
Glycine	G	0.48	−0.4	0.48	0.09
Histidine	H	−0.4	−3.2	−0.4	−2.71
Isoleucine	I	1.38	4.5	1.38	4.99
Lysine	K	−1.5	−3.9	−1.5	−3.41
Leucine	L	1.06	3.8	1.06	4.29
Methionine	M	0.64	1.9	0.64	2.39
Asparagine	N	−0.78	−3.5	−0.78	−3.01
Proline	P	0.12	−1.6	0.12	−1.11
Glutamine	Q	−0.85	−3.5	−0.85	−3.01
Arginine	R	−2.53	−4.5	−2.53	−4.01
Serine	S	−0.18	−0.8	−0.18	−0.31
Threonine	T	−0.05	−0.7	−0.05	−0.21
Valine	V	1.08	4.2	1.08	4.69
Tryptophan	W	0.81	−0.9	0.81	−0.41
Tyrosine	Y	0.26	−1.3	0.26	−0.81

Protein I: W**T**FESRNKPA**K**DP**V**ILWLNGGPGCSS**L**TGL.

Protein II: W**F**FESRNKPA**N**DP**I**ILWLNGGPGCSS**F**TGL.

**Table 2:** The slope difference distances of ND5 proteins of nine species by our approach.

	Gorilla	Pygmy	Common	Fin whale	Blue whale	Rat	Mouse	Opossum
Human	**0.2731**	**0.1965**	**0.2125**	0.7717	0.7816	0.8681	0.8075	1.5101
Gorilla		**0.2662**	**0.2753**	0.7824	0.7899	0.9509	0.8444	1.6152
Pygmy			**0.1748**	0.7747	0.7843	0.8898	0.8082	1.5345
Common				0.7588	0.7700	0.8909	0.7701	1.5315
Fin whale					**0.1077**	0.7588	0.7314	1.4427
Blue whale						0.7947	0.7452	1.4880
Rat							**0.4995**	1.4290
Mouse								1.3969

**Table 3:** The distance matrix for ND5 proteins of nine species calculated by ClustalW.

	Gorilla	Pygmy	Common	Fin whale	Blue whale	Rat	Mouse	Opossum
Human	**10.7**	**7.1**	**6.9**	41.0	41.3	50.2	48.9	50.4
Gorilla		**9.7**	**9.9**	42.7	42.4	51.4	49.9	54.0
Pygmy			**5.1**	40.1	40.1	50.2	48.9	50.1
Common				40.4	40.4	50.8	49.6	51.4
Fin whale					**3.5**	45.3	46.8	52.7
Blue whale						45.0	45.9	52.7
Rat							**25.9**	54.0
Mouse								50.8

**Table 4:** Comparison of Jackknife Accuracies of Different Classification and algorithm.

Dataset	Algorithm	Accuracy (%)				
		All-α	All-β	α/β	α + β	Overall
639 domains (25% sequence identity)	SVM [33]	73.91	61.04	81.92	33.92	62.34
	IB1 [33]	53.62	46.10	68.93	34.50	50.94
	C4.5 [33]	59.42	49.35	58.19	28.65	48.44
	Naive Bayes [33]	55.07	62.34	80.26	19.88	54.38
	Logistic regression [33]	69.57	58.44	61.58	29.82	54.06
	*k*-NN [20]	54.35	36.36	77.97	37.06	51.96
	Our method	54.71	**62.87**	72.32	**53.37**	**60.82**

**Table 5:** The other four Jackknife performance of different classification using our method.

Classes	Sensitivity (%)	Specificity (%)	MCC (%)	AUC (%)
All-α	52.97	61.40	11.64	60.93
All-β	61.36	64.89	25.97	61.63
α/β	65.25	91.58	50.36	87.51
α + β	52.14	57.14	8.21	53.51
